# Supplementary material for: How a “Smart regulatory” platform can improve the property fee adjustment dilemma
Source: PLoS One. 2023 Nov 30;18(11):e0294920. doi: 10.1371/journal.pone.0294920 (PMC10688660; doi:10.1371/journal.pone.0294920)
Supplement: Attachment — Submitted filename: Response to reviewers.docx [file pone.0294920.s002.docx]

Dear reviewer:

Thank you for your decision and constructive comments on my manuscript. We have carefully considered the suggestion of Reviewer and make some changes. We have tried our best to improve and made some changes in the manuscript. As suggested, the point-to-point revisions are described below:

Reviewer #1:

1. **Provide more background details on the specific property management context related to the study area.**

Thank you for your suggestions. We have made the corresponding modifications in the background section, added specific details:and incorporated relevant references to enhance the description and elucidation of the property management background in the research area.

Specifically:Property management part of this paper supplements the research of scholars on the status quo of the property survival environment and adds examples to support it, and the smart regulation part of this paper supplements the suggestions put forward by other scholars for the improvement of government regulation.

2. **Expand the literature review to include behavioral economics concepts like irrational biases relevant to pricing games.**

Thank you for your suggestions. In the literature review section, we have added a discussion on game theory and specifically examined the aspect of irrationality in the game process to broaden the scope of the literature review.

3. **Discuss practical implementation, adoption strategies, costs and challenges associated with the proposed smart regulatory platform.**

Thank you for your suggestions.In the theoretical analysis section of Chapter 4, we have supplemented the relevant details of "smart supervision".

The additional paragraph in the theoretical analysis section is as follows:The government plays a crucial role in community governance and should be responsible for establishing a publicly available information disclosure platform to ensure transparency in property service quality and costs. At the same time, property companies should regularly report their detailed income breakdowns and the deployment status of community staff to this platform. The government can assess the quality of property services based on this data and provide reasonable market-adjusted prices. Homeowners can use the platform to report property issues to the government, which can conveniently collect and organize these complaints, conduct regular inspections based on homeowner feedback, and impose penalties on underperforming property companies.The specific operational concept of the platform in this paper is shown in Fig 1.

4. **The Discussion needs to be a coherent and cohesive set of arguments that take us beyond this study in particular and help us see the relevance of what the authors have proposed. The authors need to contextualize the findings in the literature and need to be explicit about the added value of your study towards that literature. Also, other studies should be cited to increase the theoretical background of each of the methods used. Findings should be contextualized in the literature and should be explicit about the added value of the study towards the literature. The contribution and implications of the article are yet to be specified. Please refer to the style, DOI: 10.1016/j.renene.2023.118960 or 10.1007/s10479-023-05334-1**

Thank you for your suggestions. In the conclusion, we have combined our research to explain the value of this article in terms of theoretical contribution, practical significance, and research insights.

The Conclusion section has been revised as follows:This study introduces the "intelligent supervision" technology to address the issue of property fee adjustment, which has important theoretical, practical, and promotional value. Theoretically, this study reveals the limitations of traditional regulatory methods and proposes a new solution, enriching the research content in the field of property management. From a practical perspective, intelligent supervision technology can improve the fairness and efficiency of property fee adjustment, helping to enhance the quality of property services and owner satisfaction. From a promotional perspective, intelligent supervision technology can also be applied to other industries to address the problems of information asymmetry and regulatory difficulties. In conclusion, this study has important value in terms of theory, practice, and promotion.

5. **In the conclusion, broaden the implications of results to other pricing dilemmas like utility bills, tuition fees etc. beyond just property management.**

Thank you for your suggestions.In the conclusion, we expanded the scope of the impact of our research findings.

The additional paragraph in the Conclusion section is as follows:

The traditional regulatory approach has led to information asymmetry, which is also one of the reasons for the difficulty in adjusting property expenses. Through game analysis, we conclude that intelligent regulatory platforms can solve the problem of information asymmetry by improving transparency, thereby promoting the success of property expense adjustments. Information asymmetry is a common challenge in many industries, and the successful application of intelligent regulatory platforms in one industry can also provide similar solutions for other industries. For example, by real-time monitoring of household electricity and water consumption and sharing the data with homeowners and property management companies, intelligent regulatory platforms can avoid information asymmetry and ensure the fairness and reasonableness of expense adjustments. In summary, the application of intelligent regulatory platforms can solve the problem of information asymmetry, promote cooperation and trust among all parties, and provide important reference for solving similar problems in other industries.

6. **Carefully proofread the manuscript to fix minor grammatical errors.**

Thank you for your suggestions，We carefully proofread the manuscript and corrected any grammar errors.

Reviewer #2:

1. **It is recommended to change the subtitle to "MatLAB-based Evolutionary Game Analysis" or delete the subtitle.**

Thank you for your suggestion .We have deleted the subtitle.

2. **"with the increase in the use time of buildings, the cost of daily maintenance and repair will also increase to some extent" Should be amended to" As buildings last longer, the cost of routine maintenance increases”.**

Thank you for your suggestion. "with the increase in the use time of buildings, the cost of daily maintenance and repair will also increase to some extent" has been modified to " As buildings last longer, the cost of routine maintenance increases”.

3. **Reference 11 was published in 2013, “For example, in 2021, a district in Qingdao received nearly 7,000 complaints. Among the 237 communities under the jurisdiction of this district, 113 communities had property fees lower than 0.6 yuan, accounting for 47%." There is a problem with the annotation of the statement literature, please re-annotate or modify.**

Thank you for your suggestions.The reference literature is a summary of the phenomenon of low property fees, and this issue was raised in 2013. In order to make the argument more relevant to reality, this article uses a community case study in 2021 as a real-life example, and there is no time conflict between the two. The narrative in the article indeed had problems, causing confusion in logic, and has now been modified.

The article is now described as follows:In addition, Research has found that in situations where property service fees are low, property companies often deliberately and selectively ignore negative feedback from homeowners. This practice exacerbates conflicts between homeowners and property companies. For example, in 2021, a district in Qingdao received nearly 7,000 complaints. Among the 237 communities under the jurisdiction of this district, 113 communities had property fees lower than 0.6 yuan, accounting for 47%. Therefore, long-term low property fees are not conducive to community governance, and property fees need to be adjusted.

4. **"Keyword" should be "Keywords".**

Thank you for your suggestions.We have made the necessary revisions as requested."Keyword"has been modified to "Keywords".

Reviewer #3:

1. **In keywords, the capitalization of the first letter needs to be consistent. Please determine how to write "Evolutionary Game".**

Thank you for your suggestions.We have fixed the formatting issue with the keywords."Evolutionary Game"has been modified to"Evolutionary game"

2. **In the literature review section, please confirm whether the author of the cited literature needs to be given his or her full name, and then modify it.**

Thank you for your suggestions.We have standardized the format of references.

3. **There are many formulas in the article, which can well explain the content of the article. But some formulas contain editing errors. Please check each formula to ensure its correctness. And make sure the format of the formula is consistent and try to make it as simple and beautiful as possible.**

Thank you for your suggestions.We have modified and simplified the equations in the article according to the requirements of the journal.

4. **The labeling of the chart is a bit confusing. First of all, the first picture is named "Image 1", which does not correspond to the "figure" below. Moreover, "Figure 2" appears twice in the manuscript. Please re-layout to ensure that the order of the charts is correct and reasonable.**

Thank you for your suggestions.We have revised and aligned the tables and figures in the article to the requirements of the journal.According to the journal's requirements, the labels for the figures have been standardized. The label for the images is "Fig" and the label for the tables is "Table".

5. **The content of the chart also needs to be modified to ensure that its introduction is beautiful. Taking "Figure 1" as an example, it is best to keep the arrow sizes the same. The meanings of the abscissa and ordinate in "Figure 2" need to be clearly marked in the figure. Other charts should also be modified appropriately to ensure that readers can understand them.**

Thank you for your suggestions.The arrows in Fig 1 have been standardized. As for Fig 2 and other graphs, the X and Y coordinates have been labeled accordingly.

6. **The format of references is not unified. For example, the citation format of articles 4, 5, and 6 is obviously different from other documents. Even the author's name is not displayed in the 13th article. I don't know if it's my fault. Please follow the prescribed document citation format and carefully modify the format of the reference to ensure the uniformity of the citation format.**

Thank you for your suggestions.We have standardized the format of references.

7. **There are also some writing errors in the article, such as the capitalization of the first letter, the missing left bracket, etc. Please revise the article content carefully to avoid simple errors.**

Thank you for your suggestions.We have already made corrections to the spelling errors found in the article.
